# A Review of Wafer-Level Packaging Technology for SAW and BAW Filters

**DOI:** 10.3390/mi16030320

**Published:** 2025-03-11

**Authors:** Xinyue Liu, Wenjiao Pei, Jin Zhao, Rongbin Xu, Yi Zhong, Daquan Yu

**Affiliations:** 1 School of Electronic Science and Engineering, Xiamen University, Xiamen 361005, China; 36120221150446@stu.xmu.edu.cn (X.L.); wenjiaop2022@163.com (W.P.); xurongbin@xmu.edu.cn (R.X.); zhongyi@xmu.edu.cn (Y.Z.); 2 Institute of Electronics Packaging Technology and Reliability, Beijing University of Technology, Beijing 100021, China; zhaojin@emails.bjut.edu.cn

**Keywords:** wafer-level packaging technology, surface acoustic wave filters, bulk acoustic wave filters

## Abstract

This paper presents a comprehensive review of advancements in wafer-level packaging (WLP) technology, with a particular focus on its application in surface acoustic wave (SAW) and bulk acoustic wave (BAW) filters. As wireless communication systems continue to evolve, there is an increasing demand for higher performance and miniaturization, which has made acoustic wave devices—especially SAW and BAW filters—crucial components in the Radio Frequency (RF) front-end systems of mobile devices. This review explores key developments in WLP technology, emphasizing novel materials, innovative structures, and advanced modeling techniques that have enabled the miniaturization and enhanced functionality of these filters. Additionally, the paper discusses the role of WLP in addressing challenges related to size reduction and integration, facilitating the creation of multi-functional devices with low manufacturing costs and high precision. Finally, it highlights the opportunities and future directions of WLP technology in the context of next-generation wireless communication standards.

## 1. Introduction

In recent years, with the demand for 5G communication for cell phones, the rise in the Internet of Things, and the demand for automotive electronics, the requirements for Radio Frequency filtering technology have been increasing, prompting the rapid development of related technologies. Radio Frequency refers to electromagnetic waves that can propagate over long distances and across various spatial locations, typically within the frequency range of 300 kHz to 300 GHz. RF is widely used for wireless communication. RF system [1], a critical system of the communication infrastructure, is responsible for handling the transmission and reception of RF signals. This includes the transceiver, antenna, and RF front end. The RF front-end system comprises a transmitter, antenna, and receiver path, with the transmitter and receiver paths collectively forming the RF front end. Specifically, the transmitting path typically includes components such as the power amplifier and duplexer, while the receiving path includes elements like the antenna tuner, filter, low-noise amplifier (LNA), and switch.

Filters are essential in the RF system because they process the interference noise generated during the signal reception process across different frequency bands [2]. The performance of these filters directly impacts the quality of wireless communication. Currently, RF filters are mainly categorized into LC filters and piezoelectric filters, of which LC filters are mainly low-temperature co-fired ceramic process filters and integrated passive component filters; piezoelectric filters include surface acoustic wave filters and body acoustic wave filters. Piezoelectric filters utilize the characteristics of piezoelectric materials to filter RF signals with high frequency stability and excellent selectivity. Surface acoustic wave filters utilize the property of surface acoustic waves propagating on the surface of piezoelectric materials, and have smaller size and high frequency characteristics. SAW filters usually have good temperature stability and high selectivity. BAW filters utilize body acoustic waves propagating inside the piezoelectric material, providing a higher frequency range and lower insertion loss, making them suitable for high-frequency and high-data-rate applications [3]. As communication standards continue to evolve, the number of acoustic wave devices integrated into mobile phones is steadily increasing, and this trend shows no sign of abating in the near future [4,5,6,7,8,9,10].

**Figure 1:**
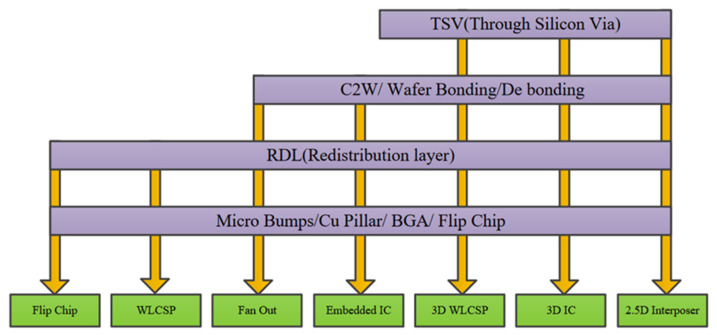
Advanced packaging processes and technologies they involve [11].

Due to the persistent miniaturization trend of filters, packaging efforts have become increasingly crucial. Wafer-Level Packaging can not only achieve high performance and multi-functional integration of devices, but also take full advantage of the characteristics of mass production using wafer-level processes, such as low cost, high-precision manufacturing, and the realization of small-sized packaging. Among them, the main processes of wafer-level packaging technology include Micro-Bump processing, rewiring, ball placement, temporary bonding/debonding, through-silicon via (TSV) interconnection processes, and so on. Advanced packaging technologies need continuous innovation and development to meet the more complex requirements of three-dimensional (3D) integration. As shown in Figure 1, it demonstrates the relationship between advanced packaging processes and the technologies they involve [11], most of which are related to wafer-level packaging technologies. For example, 3D WLCSP, 3D IC, and 2.5D interposer structures all utilize TSV technology.

This paper provides a comprehensive review of the significant advances in wafer-level packaging technology for surface acoustic wave and bulk acoustic wave (BAW) filters in recent years, which have contributed to the continued evolution of this field. Key developments discussed include novel structures, innovative materials, advanced modeling techniques, and ongoing size reduction facilitated by the introduction of new packaging methods. The paper begins with an overview of the historical development of wafer-level packaging technology, followed by a detailed review of its application in SAW and BAW filters, respectively. Finally, the paper concludes by summarizing the opportunities and challenges associated with wafer-level packaging for filters, offering insights for future research directions.

## 2. IC Packaging Technology

Since the commercialization of silicon transistors in the 1960s, semiconductor chips have found widespread applications in fields such as computers, communications, aerospace, and industrial electronics, significantly enhancing the convenience of everyday life. The development of semiconductor chips has directly or indirectly driven societal progress. Typically, the chip production process consists of two main stages: the Front End of Line (FEOL) and the Back End of Line (BEOL) processes [12,13]. The FEOL process primarily focuses on manufacturing transistor-related structures, while the BEOL process is responsible for establishing connections between transistor multilayer conductive vias, metal interconnects, and external interfaces. Together, these processes enable the chip to perform its intended functions. Upon the completion of the chip, the packaging process is required to facilitate interconnection between the chip and the system, providing power distribution, signal routing, heat dissipation, mechanical support, and protection. These functionalities are critical to meet the demands of portable electronic devices, which require ultra-lightweight, ultra-thin, high-performance, and low-power consumption features. The five essential functions of a package can be summarized as follows [11]:

1. Signal Distribution: Serves as an electrical interconnection channel between the device and the circuit board, distributing signals from the high-density chip’s pad area to a larger spatial region.
2. Power Distribution: Facilitates the distribution and conduction of power/voltage between the device and the circuit board.
3. Heat Dissipation Pathway: Transfers heat generated during the device’s operation to the external environment, such as using heat sinks to ensure that the device operates within a tolerable temperature range.
4. Mechanical Support: Provides sturdy and reliable mechanical support for the device.
5. Device Protection: Shields the device from external factors such as electromagnetic interference, moisture, dust, and vibrations, thus enhancing the reliability and lifespan of the device.

**Figure 2:**
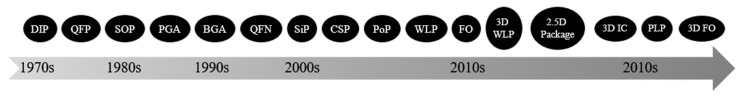
Evolution of advanced packaging technologies.

As shown in Figure 2, the development trend of major packaging technologies can be characterized by the following key aspects [14,15,16,17]: 1. transition from single-component packaging to packaging systems; 2. evolution from single-chip to multi-chip configurations; 3. shift from flat packages to (3D) packages; 4. the transition from traditional interconnection methods to flip-chip interconnection and through-silicon via (TSV) technology as the primary interconnection techniques.

To reduce transistor size and optimize semiconductor chip performance and power consumption, the industry is adopting new structures (e.g., strained silicon [18,19]), new materials (e.g., graphene [20,21]), and advanced fabrication methods (e.g., double-exposure [22,23]). However, these innovations are expected to significantly increase research, development, and manufacturing costs. Moreover, only a few companies globally can afford the R&D and mass production of the most advanced process nodes, such as TSMC (Hsinchu, Taiwan), Samsung (Gyeonggi-do, Republic of Korea), and Intel (Santa Clara, CA, USA), which are the leading players in the development of 5/3 nm processes.

**Figure 3:**
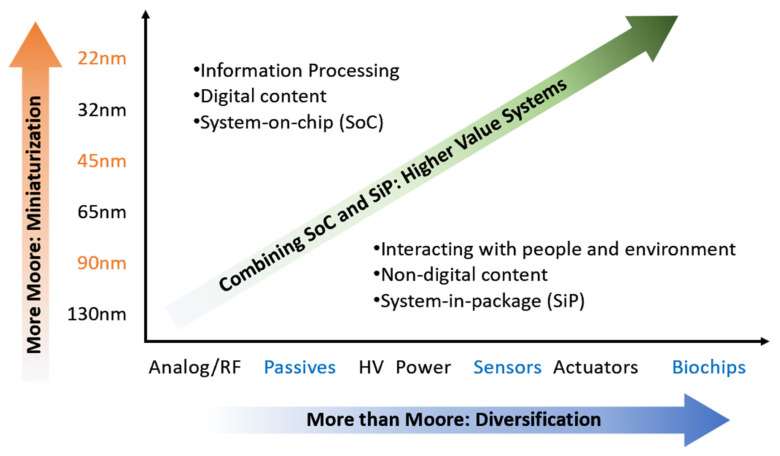
Evolution of semiconductor technology according to Moore’s law [27].

According to the international semiconductor technology development roadmap [24,25], IC technology continues to follow Moore’s law by advancing towards high-value, multi-type, and multi-functional designs. As shown in Figure 3, 3D packaging based on stacked interconnect integration is a key research direction for sustaining Moore’s law. This approach involves vertically stacking multiple chips or systems, such as image sensors, RF chips, and memory modules, to achieve more diversified functionality and enhanced system intelligence [26].

Through an overview of the evolution of IC packaging technology and the packaging technology required in the post-Moore era, and in the context of the era of rapid economic development driven by scientific and technological innovation, it is made clear that IC is an emerging strategic technology that will lead the future. To benchmark the development of international and domestic multi-application scenarios in the context of the 5G era, an important part is the need to promote the development of RF front-end chips in the direction of modularization, ecology, and diversification [28]. Among them, RF filters are the most important discrete devices in RF front ends, and they are present in almost all RF module products, occupying most of the market share of RF front ends.

## 3. Research Progress in Wafer-Level Packaging of SAW Filters

### 3.1. Fundamentals of SAW Filters and Packaging Requirements

**Figure 4:**
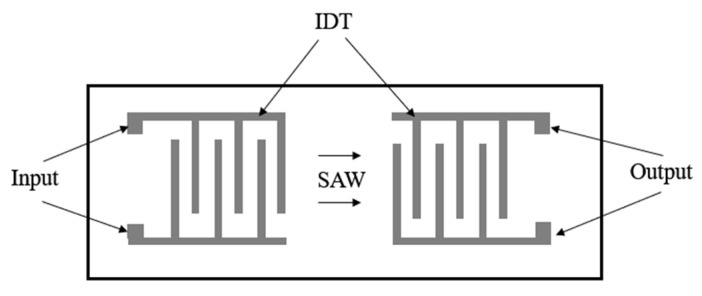
Schematic of SAW filter [29].

Surface acoustic wave is a means of realizing the transmission of acoustic waves along the surface of a solid. A SAW filter consists of piezoelectric materials and two Interdigital Transducers (IDT), which are also referred to as the device functional areas in packaging, as illustrated in Figure 4. The core role of IDT is to achieve the conversion between acoustic wave signals and electrical signals [29]. The conversion principle is as follows: When IDT is deposited on the surface of piezoelectric materials and receives a voltage signal, it causes the piezoelectric materials to generate mechanical pressure, resulting in a change in the distance between atoms within the piezoelectric crystal and breaking the original balance of positive and negative charges. In the vertical direction, the amplitude of the acoustic wave will decay rapidly, and the generated acoustic wave signal mainly propagates along the surface of the piezoelectric substrate. However, the IDT at the output end receives the acoustic wave in the horizontal direction and converts it into an electrical signal for output. Commonly used piezoelectric materials include lithium tantalate (LiTaO_3_, LT) and lithium niobate (LiNbO_3_, LN), etc.

The frequency of the propagation of the SAW can be obtained by the following equation [4]:

F=V÷λ

*V* represents the propagation speed, and λ is the spacing between the IDT electrodes. As the electrode spacing gets smaller, the frequency of the SAW gets higher. When the electrode spacing is small, it will lead to electron migration and heat generation problems. Therefore, it is necessary to compensate for the temperature on the IDT surface, and usually, a dielectric layer is added to the IDT to increase the stiffness at elevated temperatures to improve the filtering characteristics, which is known as a Temperature-Compensated SAW Filter. As a result, a filter with low insertion loss over a wide bandwidth and good out-of-band rejection near the passband can be designed by using a complex IDT layout.

For filters above 2 GHz, the IDTs are typically made of aluminum electrodes with a thickness of 150 nm and a width of less than 1 μm. Therefore, for surface wave propagation, the package must provide a cavity structure above the functional area of the device that protects and prevents the corrosion of the IDTs. In order to test the corrosion resistance of the package, high temperature/high humidity reliability tests are required. Meanwhile, SAW filters are used in different scenarios where the package must withstand different temperature variations and needs to pass temperature cycling or thermal shock reliability tests. In addition, small size, competitive package processing cost, and maintaining excellent reliability and electrical performance of the package are all requirements for packaging solutions.

This section provides an overview of the evolution of SAW device packaging schemes over the last 20 years.

### 3.2. Evolution of Ceramic-Based Packaging Technologies for SAW Filters

**Figure 5:**
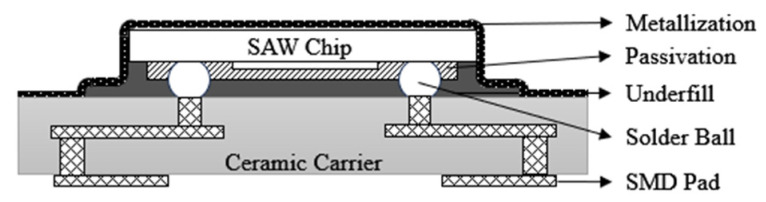
Schematic of CSSP1 [31].

As early as 2000, EPCOS (now RF360 Europe Ltd, Munich, Germany.) released the first generation of chip-sized SAW filter packages(CSSP1) [30,31], as shown in Figure 5, with the main feature being the use of flip-chip soldering technology. The package has evolved from the traditional wire bonding method to the flip-chip soldering technique, which realizes the shortening of signal lines and improves the electrical interconnection performance of the device. Nowadays, SAW filter packages are pursuing the requirements of small size and miniaturization, and the minimum package area realized by CSSP1 technology is 2.0 mm × 2.0 mm, which does not meet the current miniaturization requirements and has faded out from the market.

**Figure 6:**
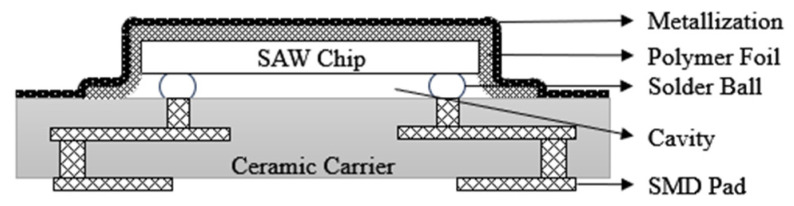
Schematic of CSSP2 [30].

In order to achieve a smaller size, a second-generation CSSP2 was developed in 2002 [30], as shown in Figure 6. The SAW filter uses lead-free SAC solder ball bumps soldered to High-Temperature Co-fired Multilayer Ceramics (HTCC) or Low-Temperature Co-fired Ceramics (LTCC) with a ball diameter of 100 μm and a 125 μm under bump metallization (UBM). A polymer film is used to cover the backside of the flip-chip device, forming a cavity structure in the active area of the SAW filter and enabling good adhesion between the film and the chip and substrate to ensure that there is a certain gap between the chip and the substrate so that the device can work properly. Then, the laser is used to remove the film layer covering the edge of the SAW filter, followed by sputtering and electroplating processes to deposit Cu and nickel (Ni) layers to prevent moisture from spreading to the inside of the package, and finally cutting the ceramic substrate to separate the device.

**Figure 7:**
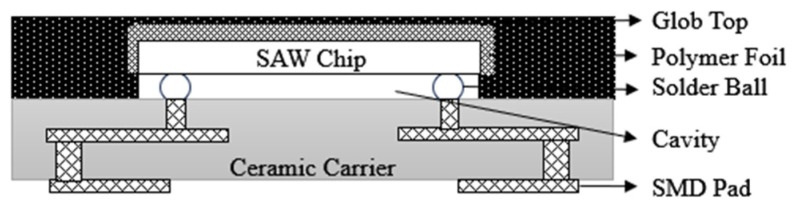
Schematic of CSSP3 [30].

EPCOS’ third-generation SAW filter CSSP3 packaging technology [30], shown in Figure 7, also uses flip-chip soldering to interconnect the SAW filter to the HTCC substrate compared to the previous two generations, but achieves a smaller solder ball size and a smaller package thickness based on CSSP2. At the same time, this process molds the backside of the SAW filter and ensures that the cavity between the chip and the substrate is not filled by selecting a specific molding compound. Considering the non-airtight nature of the plastic encapsulation material, moisture can diffuse into the active area of the chip. Therefore, the IDT area of the SAW filter samples to be encapsulated should be coated with a layer of inorganic passivation layer, such as silicon dioxide, to prevent IDT corrosion. At the same time, silicon dioxide has a negative temperature coefficient, and when the temperature rises, silicon dioxide contraction can maintain the original shape of the LT chip, thus ensuring the stability of the device.

**Figure 8:**
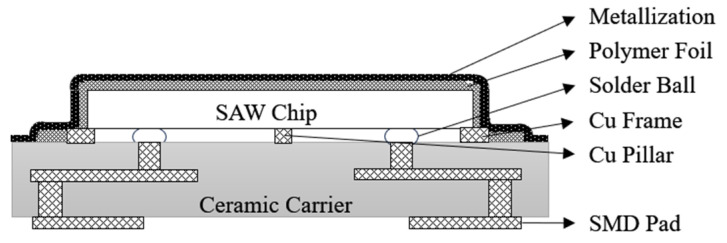
Schematic of CSSP3 Plus [30].

Based on the non-hermetically sealed CSSP3 package scheme, EPCOS has developed a hermetically sealed package technology, CSSP3 plus [30], as shown in Figure 8. A combination of photolithographic and electrochemical processes is used to photolithographically plate a copper pillar frame on a ceramic substrate and then flip-chip the SAW filter onto the ceramic substrate. After soldering, similar to the CSSP2 molding process, the film is applied first and then encapsulated, and then the excess film layer is removed at the edges by laser and the Cu/Ni layer is plated for hermetic encapsulation. The development of a series of CSSP processes by EPCOS has brought the entire flip-chip film encapsulation process to the extreme and has played a crucial role in the structural stability and high reliability of the devices.

### 3.3. Emergence of Wafer-Level Packaging (WLP) Solutions

With the further development of the technology, SAW filters have gradually adopted wafer-level package technology, which can realize smaller sizes and thinner devices.

In 2006, S. Gao et al. proposed a SAW filter wafer-level packaging structure based on the metal bonding process and analyzed the reliability of the packaging structure through the three-dimensional thermomechanical finite element method (FEM) [31]. The cover wafer needs to complete micro-vias, electroplating, grinding, polishing, and thinning processes, and the surface acoustic wave wafer needs to complete the IDT functional area, metal pads, photoresist patterning, and solder deposition processes. Then, the two wafers are bonded through the wafer bonding process to realize the packaging structure. And, through simulation, it is concluded that the coefficient of thermal expansion (CTE) is the parameter that affects the maximum stress of the packaging structure. J. L. Pornin et al. proposed a thin-film packaging technology to realize the development of SAW/BAW filter packages in 2007 [32,33,34]. The cavity structure required for the filter package is made of a polymer sacrificial layer material. A cap layer, such as silicon dioxide, is deposited on the surface of the SAW/BAW filter wafer by chemical vapor deposition, and the sacrificial layer releases vias and device outputs are dry-etched. The sacrificial layer material is removed by dry-etching. After that, another polymer material is spin-coated and photolithographed to expose the I/O terminals, which are then electrically interconnected by a plating process to complete the device package.

However, with the advent of a dry film photoresist (DFR) material, K. Sakinada et al. [35,36] applied it to SAW filter packages. A two-step dry film photoresist lamination process was used to form the cavity structure required for SAW filter packages without the use of sacrificial layer materials. First, a wall structure surrounding the IDT region of the SAW filter was created by laminating the photoresist material on the surface of the SAW filter wafer, and another dry film photoresist layer was applied on top of the wall to form a cavity structure on top of the IDT. For this packaging idea, photoresist is a very promising material for acoustic filter packaging, but considering that the adhesion between the resist layer and the piezoelectric substrate will result in delamination between the two, which will lead to device failure in the subsequent packaging processing and/or packaging-level reliability tests. At the same time, there may be outgassing of the dry film photoresist material at elevated temperatures, causing the erosion of the IDT structure. Moreover, in RF module integrated packaging, high temperature/high pressure can also degrade the mechanical strength of the molded cavity structure, causing it to collapse and rendering the SAW filter inoperable. Therefore, to address these failure behaviors, different experts and scholars in the industry have carried out a lot of research work trying to promote the continuous development of polymer semiconductor materials, and J. H. Kuypers et al. [37] and Nao Honda et al. [38] used polymer cavity packaging to achieve lower packaging cost and ultra-thin package thickness, which has the advantages in terms of weight and size. Typically, in the SAW/BAW filter cavity package, SU-8 3000CF(Nippon Kayaku Co., Ltd. is based in Tokyo, Japan) dry film resist is used as the wall and cap layer structure in the package.

**Figure 9:**
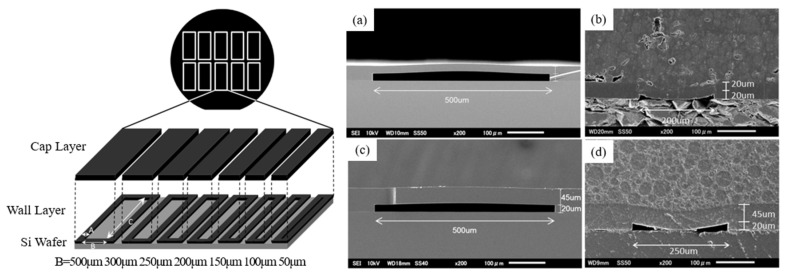
Collapse test at high potting pressure [38]: (**a**) Cross section SEM image (Cap thickness/Cavity width: 20 μm/500 μm); (**b**) Cross section SEM image of 45 μm thick cap after molding test(200 μm cavity) (**c**) Cross section SEM image (Cap thickness/Cavity width: 45 μm/500 μm); (**d**) Cross section SEM image of 45 μm thick cap after molding test (250 μm cavity).

As shown in Figure 9 [38], the adopted DFR package technology with a cap layer thickness design of 20 μm or 45 μm, and cavity widths of 200 μm and 250 μm, respectively, collapsed under high potting pressure (9 MPa, 175 °C). Thinner DFR thickness helps to achieve lower cavity packages, but the increase in cavity size with lower DFR thickness also results in a pitted or collapsed cap layer, as seen in the industry when cavity length and/or width are designed to exceed 500 μm.

### 3.4. Advanced WLP Architectures for Miniaturization

**Figure 10:**
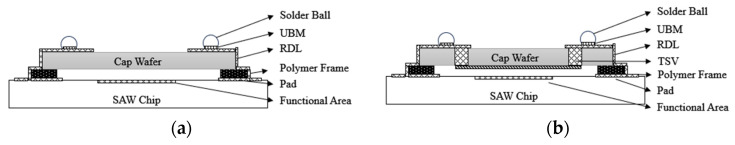
Schematic of DSSP: (**a**) SAW filter wafer in DSSP; (**b**) SAW filter wafer with inductor in DSSP [39,40].

RF360 has introduced the Die-Sized SAW Packaging (DSSP) technology [39,40,41] which means that the package footprint is the same as the chip size, realizing a true chip size package. As shown in Figure 10a, two LT or two LN wafers are wafer bonded by adhesive to create a cavity structure. The first wafer (i.e., device wafer, SAW filter wafer) carries the SAW device structure and forms the bonding framework by patterning the polymer adhesive on its surface through photolithography. In addition, the second wafer (i.e., Cap Wafer) is optically aligned and bonded to the first wafer, and the cap layer is separated by reverse grinding and cutting only the cap layer. Subsequently, the interconnect layer was realized by surface redistribution layer (RDL) DFR technology to cover the device input/output electrode surfaces, the sidewalls of the bonding layer and the cap layer sidewalls, and part of the top surface of the cap layer, and the final package is shown in Figure 10. The DSSP solution was realized entirely on the wafer, and the cap layer was proved to be able to withstand the collapse test with high pressure (about 5 MPa) by the post molding test.

Based on the remaining variants of DSSP technology [41], as shown in Figure 10b, solutions with integrated inductors have been proposed to improve chip integration and provide some advanced solutions for RF module applications. However, the disadvantage is that the cap layer thickness is usually 100~400 μm, which does not realize the thinner package thickness.

**Figure 11:**
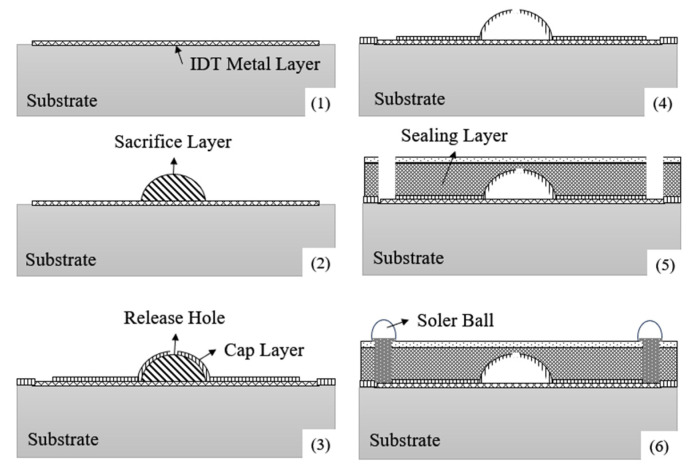
Packaging process flow based on sacrificial layer technology: (1) IDT formation; (2) sacrifice layer formation; (3) release hole and cap layer formation; (4) release of the sacrificial layer; (5) sealing layer and vias formation; (6) solder balls formation [42].

In addition, in order to further reduce the height of the package and to obtain a package solution with high mechanical stability and excellent electrical performance that can be integrated into a module, RF360 has developed a new package technology, the Thin-Film Acoustic Package (TFAP) [42], which is suitable for standard SAW filters in 2015, as shown in Figure 11. The process consists of coating the device surface with a sacrificial layer material to form a dome-shaped bump to avoid sharp edges that may reduce mechanical stability. A first dielectric layer (i.e., cap layer) is then deposited on top of the sacrificial layer structure, and small holes are created in the device pads and above the sacrificial layer for external interconnections and sacrificial layer removal. After removal of the sacrificial layer by dry-etching, the polymer covers the first dielectric layer and the same lithography opens only at the pads, making the entire device structure hermetically sealed. In order to stabilize the mechanical structure of the package, a second dielectric layer (i.e., the reinforcement layer) is deposited on top of the sealing layer and photolithographed open at the pads for subsequent electrical interconnections.

**Figure 12:**
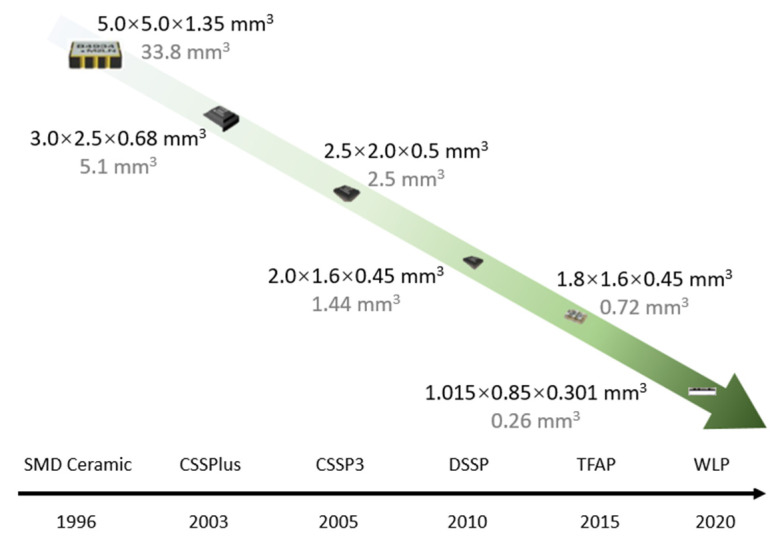
Evolution of SAW packaging and miniaturization trends [43].

The RF filter is available in the TFAP solution, which is one of the thinnest packages available for direct RF module integration. It meets the current market requirements for SAW packages while also meeting stringent performance specification tests. The evolution of SAW packaging and miniaturization trends are illustrated in Figure 12 [42,43].

**Figure 13:**
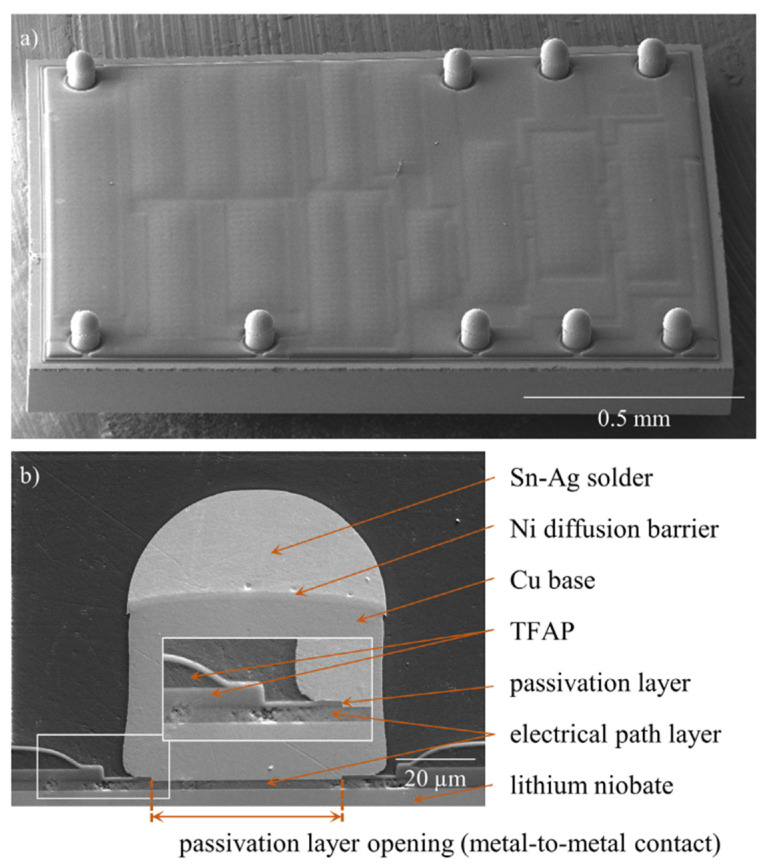
(**a**) SEM image of a fully processed SAW RF-filter chip with CPBs and (**b**) cross-sectional SEM image of a CPB. The area of the white frame is magnified in the inset. The peculiarities in the electrical path layer are caused by cross-section preparation [46].

The standard TFAP packaging technology uses solder bumps as the interconnection interface between the flip-chip and the printed circuit board (PCB). When the SBs are soldered to the PCB, a certain gap is needed between the solder ball and the PCB for polymer underfill, and with the miniaturization of the SAW filter structure, the size of the SBs needs to be further reduced while the unit area occupied by the solder ball remains unchanged, which will make the polymer underfill gap smaller and the underfill more difficult. J. Schober et al. [44,45,46,47] used copper pillar bumps (CPBs) as the interconnection method of the RF filter flip-chip, as shown in Figure 13, which can realize a further reduction in the device package size. This can further reduce the size of the device package and achieve miniaturization. CPBs, consisting of copper posts and Ni/Sn (optional)/Ag solder caps, can be interconnected with smaller spacing and smaller chip area than SBs, which provides more freedom in RF filter design. J. Schober et al. [44,45,46,47] replaced the SBs interconnections with CPBs in TFAP and verified it in SAW filter packages. They showed the direction of the miniaturization of the SAW filter package and combined them with the conditions of consumer electronics reliability tests, such as the Temperature Cycling Test, Un-biased High Accelerated Temperature and Humidity Stress Test, and Un-biased High Accelerated Temperature and Humidity Stress Test (uH), and so on, which proved that the combination of CPBs interconnections and TFAPs can realize highly reliable packages.

In 2022, Z. Chen [48] proposed a SAW WLP method that uses a non-photosensitive dry film as a cover layer using a dry film lamination method. An organic thin layer as a support structure is constructed on a substrate, and at the same time, the vias and cavity patterns are produced by photolithography. The non-photosensitive dry film is then laminated on the support structure to form a cover layer, thus forming the desired cavity structure. Laser drilling is then used for patterning to obtain electroplated through holes, followed by subsequent processes such as electroplating, RDL, and etching. The combination of non-photosensitive dry film and laser drilling effectively simplifies the process and proposes a new solution for SAW WLP. W. Pei [49] drew on the above solution and applied a new non-photosensitive dry film with a higher Young’s modulus at high temperatures to SAW WLP. Experiments have shown that the dry film material has excellent pressure resistance at high temperatures and is cost-effective. At the same time, based on the simulation results, the study determined multiple parameters of the packaging structure and successfully applied them to actual production.

**Figure 14:**
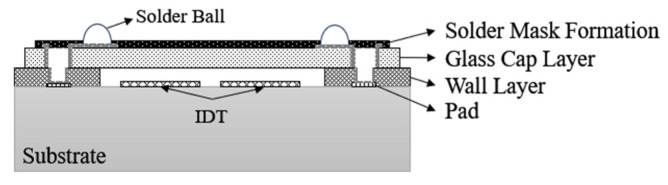
The cross-sectional schematic of WLP for SAW filter with TGVs [51].

Wang et al. [50] proposed a method of bonding a silicon substrate to a surface acoustic wave wafer by bonding materials and forming a closed cavity structure to protect the surface acoustic wave chip. A layer of photoresist is coated on the substrate, and after the cavity area is obtained through photolithography and development processes, bonding materials are attached to the substrate, and then the silicon substrate and the surface acoustic wave wafer are precisely aligned and bonded by a wafer bonder, and then the bonding material is cured by heating and pressurizing to obtain a sealed packaging structure. As shown in Figure 14, Chen et al. [51] proposed a method of using a glass cover plate to realize the SAW filter WLP pair structure. After a thin glass with a through-glass via (TGV) is formed by laser-induced chemical etching, the prepared glass cover plate mold is bonded to the corresponding SAW filter substrate to provide a safe environment for the device’s functional area to prevent it from corrosion. This technology can prevent the packaging material from degassing at high temperatures, which may cause IDT contamination.

## 4. Research Progress in Wafer-Level Packaging of BAW Filters

### 4.1. Fundamentals of BAW Filters and Packaging Demands

**Figure 15:**
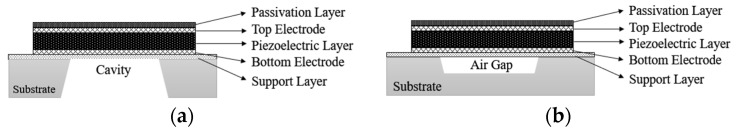
Schematic of FBAR filter: (**a**) cavity-type FBAR; (**b**) air gap-type FBAR [52].

**Figure 16:**
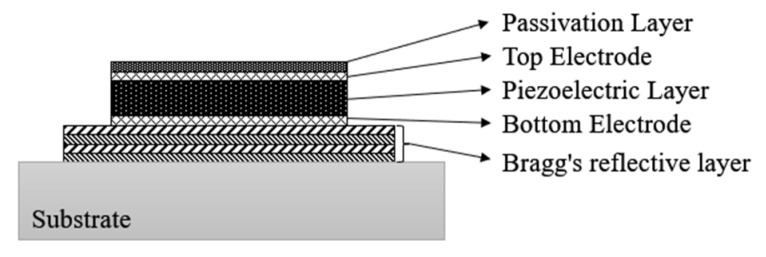
Schematic of SMR-BAW filter [52].

BAW devices include BAW resonators, filters, duplexers, etc. The BAW resonator is the heart of the rest of the devices. The basic structure of a BAW resonator is a piezoelectric film sandwiched between two metal electrodes. Based on the bulk acoustic wave theory, the BAW resonator operates by oscillating acoustic waves in the piezoelectric film, which resonates in the perpendicular direction to achieve electrical frequency selection. Multiple BAW resonators are constructed in a certain pattern, such as a trapezoidal or lattice pattern, to form a low-loss, high-rectangularity filter device. Depending on the structure, BAW resonators can be classified as either thin-film bulk acoustic resonators (FBARs), Solidly Mounted Acoustic Resonators (SMARs), or Solidly Mounted Resonator (SMR) [52,53], as shown in Figure 15 and Figure 16. Among them, the cavity-type thin-film bulk acoustic resonators are also classified into air-gap and silicon reverse-etched types based on the cavity composition.

The structure of a BAW resonator consists of an upper/lower electrode, a piezoelectric layer, and an air cavity or a Bragg layer. The difference between these two types of resonators is that the air cavity-type resonator utilizes the near-zero acoustic impedance of the air as an acoustic boundary, which results in a total reflection of the acoustic wave, thus minimizing the loss of energy. Solid-state reflective resonators use multiple layers of alternating high-impedance and low-impedance layers to form a Bragg reflective layer. Both high-impedance and low-impedance layers cause a total reflection of the incident wave, so the Bragg reflective layer is able to confine the acoustic energy to the piezoelectric stack. Due to the low mechanical strength of piezoelectric thin-film materials, they are susceptible to contamination, corrosion, and structural failure under packaging and real-world service conditions. Therefore, BAW device packaging must provide a cavity structure on the chip surface to protect against moisture and the corrosion of the piezoelectric layer. Currently, Chip Scale Packaging (CSP) and WLP are the main technologies used in the industry. In this section, the packaging solutions for body acoustic wave devices in the past 20 years are described, and the advantages and disadvantages are analyzed in order to lay the foundation for the BAW filter packaging solution proposed in this project, and provide a reference for subsequent productization.

### 4.2. Early-Stage Packaging Technologies for FBAR Filters

**Figure 17:**
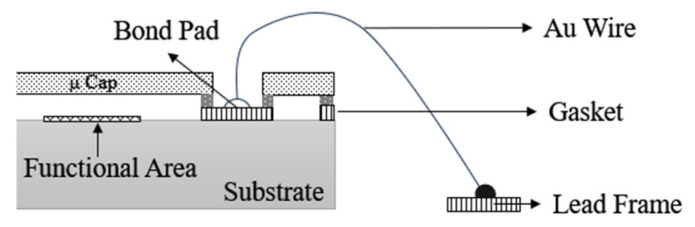
Micro-Cap package for FBAR filter [55].

As early as 2002, Agilent Technologies Inc.’s R. C. Ruby et al. [54] used a Micro-Cap wafer-level packaging scheme to realize the FBAR filter. As shown in Figure 17, the Micro-Cap structure is realized by a wafer-level process, and a W2W bonding technique is used to bond the FBAR filter wafers to the Micro-Cap wafers. After bonding, the Micro-Cap wafers are thinned using conventional wafer thinning techniques to expose the FBAR filter interconnect structure, which is then interconnected to the external circuits via wire bonding. The size of the entire package structure is large, such as 2.0 mm × 2.5 mm BAW duplexer using wire bonding to realize the interconnection with external circuits, in which the wire bonding area accounts for about 40%; if the flip-chip soldering method is used instead of wire bonding, the size of the package body can be reduced by 36% [55], along with the high cost of wire bonding processing, which restricts the competitiveness of its market.

**Figure 18:**
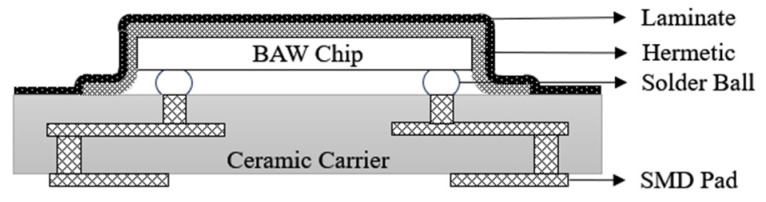
Flip-chip package for BAW chip [56].

Meanwhile, in 2003, Marksteiner S. et al. [56,57] adopted a similar process to the SAW filter CSSP2 package, as shown in Figure 18, where a flip-chip solder ball is fabricated on the front side of the FBAR filter without any additional front protection, and the backside of the FBAR filter is encapsulated with plastic sealing compound on the LTCC substrate to form a cavity structure. The front side of the FBAR filter is molded onto the LTCC substrate. This method requires the FBAR filter wafer to be cut into single chips after the backside solder ball is completed and then soldered in flip-chip mode. Since the cavity above the resonator has not been formed in advance, the high-pressure water jets required during the cutting process will damage the FBAR film structure to a certain degree, resulting in the failure of the encapsulation body.

### 4.3. Polymer-Based Non-Hermetic Packaging Solutions

**Figure 19:**
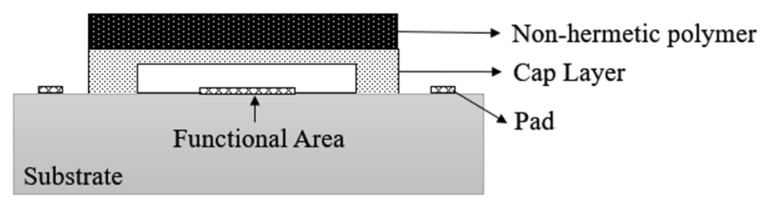
Non-hermetic polymer packaging scheme [57].

However, with the development of packaging materials and equipment, Infineon Technologies AG M. Franosch et al. [34] proposed a non-hermetic polymer packaging scheme to realize the BAW resonator package, as shown in Figure 19. Compared with the hermetic Micro-Cap chip bonding package, the width of the required sealing ring is greatly reduced, thus realizing a chip-size package structure. However, due to the solvent evaporation or outgassing of the polymer material during high-temperature curing, the resulting impurities adhere to the resonator surface, affecting its resonance frequency and leading to impaired device functionality [58].

**Figure 20:**
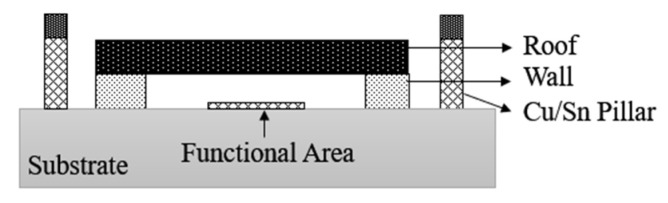
SMR-BAW filter 3D packaging solutions [59].

With the continuous updating of technology, the market demand for BAW filters is low price and small size to meet the demand for miniaturization. There are two main starting points: first, from the design side, process development drives the continuation of the miniaturization trend of BAW filters. The second is to maintain the miniaturization trend through advanced packaging technologies. In 2013, Gernot Fattinger et al. [55,59] adopted a wafer-level SMR BAW filter packaging scheme, which uses photosensitive epoxy to create a cavity above the functional area of the device, similar to the SAW filter WLP proposed by K. Sakinada et al. [35], and builds a wall and cap layer structure in the active area of the device. As shown in Figure 20, the wall and cap layer structures are created in the active area of the device, and the cavity structure is thermally cured to withstand the collapse of the cavity caused by the high-pressure molding process. Finally, copper bumps are formed by standard wafer-level processes, i.e., seed layer sputtering, photolithography, electroplating, and deboning etching, so that the final copper bump exceeds the cavity height for the subsequent flip-chip filling. However, this scheme is not applicable to air-gap-shaped FBAR filters because the air cavity region of the electrode at the bottom of the resonator has already been formed, which will cause the air cavity of the resonator region to collapse during the lithographic patterning process of the wall structure, and there is a possibility of contamination of the resonator region by the drug entering from the release holes of the sacrificial layer in the lithographic development process [60].

### 4.4. Advanced Wafer-Level Hermetic Packaging Architectures

**Figure 21:**
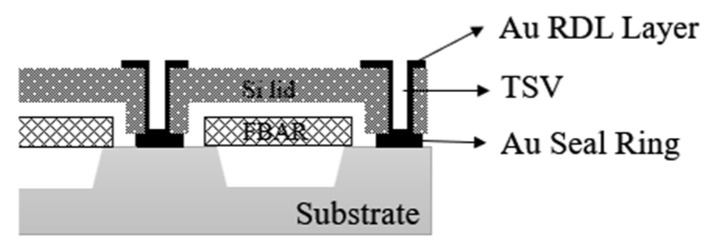
Avago Technologies’ packaging solutions for air-gap FBAR filter [61].

For the packaging of air-gap FBAR filters, Martha Small et al. [61] of Avago Technologies have realized an air-tight, high-reliability package for FBAR filters using a wafer-level packaging scheme where the FBAR filters are processed on silicon wafers to form the resonator structure, the cavity structure below the resonator, the Au signal path, and the sealing ring. The cap wafers are made from the same high-resistance silicon as the device wafers, and the cap wafers are processed using a standard micromachining process to form the TSV structure, the recessed air cavity above the resonator, and the Au Micro-Bumps. The silicon cap wafers are bonded to the FBAR filter chip using W2W bonding to achieve Au-Au hot-press bonding, and the TSV holes and surface pads are patterned to realize the electrical interconnect channel with the external circuitry. Figure 21 illustrates Avago Technologies’ wafer-level packaging solution for air-gap FBAR filters. The use of high-resistance silicon cap wafers and Au-Au hot-press bonding ensures hermetic sealing, which is critical for protecting the piezoelectric layer from moisture and corrosion. The through-silicon vias (TSVs) provide electrical interconnections, enabling high-performance operation in high-frequency applications such as 5G.

Stephen R. Gilbert et al. [62] also integrated active circuits on a high-resistance silicon cap layer to achieve integration with an FBAR filter. This solution is the thinnest package structure (thickness: 207 μm, excluding solder balls) that can be realized in the current public information, but using high-resistance silicon as the cap layer, the TSV production process is complicated, which requires photolithography development first, and then deep reactive ion etching at the photolithography openings to form the required size of the blind holes; after the formation of the blind holes, it is necessary to remove the photoresist on the surface, which will lead to the introduction of some of the impurities in the decontamination liquid into the holes, resulting in the risk of subsequent plating voids in the holes. Since silicon is a semiconductor material, an insulating layer, usually silicon dioxide, must be applied to the sidewalls of the hole. The uniform coverage of the oxide layer along the sidewalls of the vias will further increase the process difficulties. In addition, the processing of Au-Au hot-press bonding on ultra-thin wafers (97.5 μm in this case) poses a risk of cracking on 8-inch wafers (the current industry mainstream design rule for wafer-level metal bonding on 8-inch wafers and above guarantees a wafer thickness of more than 500 μm). Gold vaporization and gold-gold bonding on the surface will also result in a costly packaging process.

**Figure 22:**
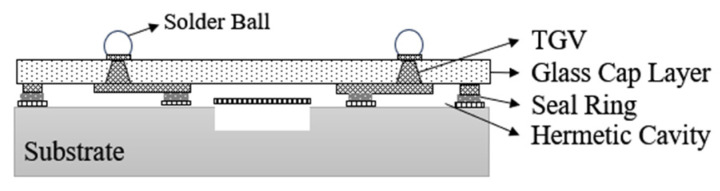
A packaging solution with TGV for FBAR filter [63].

**Figure 23:**
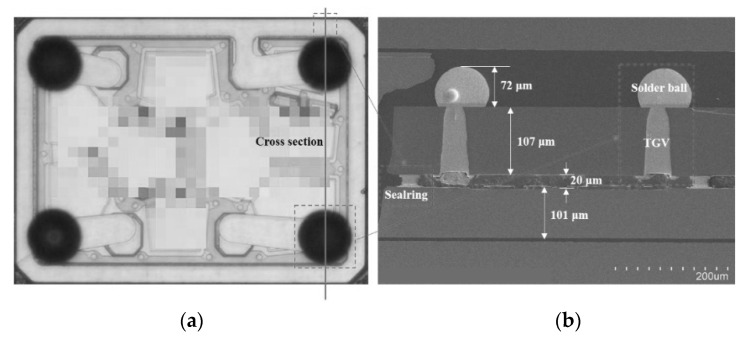
The RF filter package with TGVs after solder ball formation. (**a**) The OM picture of the RF filter package. (**b**) The cross-section view of the RF filter WLP with the solder ball [63].

Chen Z. et al. [63] address the growing demand for high-frequency FBAR filters in 5G mobile communications by proposing an innovative packaging solution using through-glass vias. This approach significantly enhances the manufacturing performance and cost-effectiveness of FBAR filters. As shown in Figure 22, the TGV fabrication process not only achieves high precision in processing but also offers substantial economic benefits. Experimental data indicate that this packaging solution exceeds industry standards in both shear strength and electrical performance, demonstrating its reliability and effectiveness in practical applications. These advantages position the TGV-based FBAR filter packaging technology as highly promising for consumer electronics, particularly in the development of future communication systems, especially 5G wireless networks. This solution provides valuable insights for the miniaturization and high-performance design of RF filters. Figure 23 shows the RF filter package with through-glass vias (TGVs) after solder ball formation. The TGVs, fabricated using laser-induced chemical etching, offer superior electrical performance and thermal stability compared to traditional through-silicon vias (TSVs).

## 5. Discussion

This review of advancements in wafer-level packaging for RF filters highlights both the significant progress and persisting challenges. While innovations in WLP have led to improved electrical and mechanical performance, ongoing issues such as the trade-off between miniaturization and performance integrity remain critical. As RF filters shrink in size, ensuring operational efficiency becomes more complex. The use of advanced materials, including polymers and ceramics, holds promise but presents challenges in high-volume manufacturing, particularly concerning moisture ingress and thermal stability. Hermetic sealing techniques have been explored, but inconsistencies in protocols and environmental testing conditions affect reliability and longevity.

The wafer-level packaging (WLP) of SAW and BAW filters has evolved significantly to meet the demands of miniaturization, high performance, and cost-effectiveness in wireless communication systems. For SAW filters, early ceramic-based packaging (e.g., CSSP) was replaced by advanced WLP techniques such as metal bonding, thin-film encapsulation, and polymer-based cavity structures. These methods enabled smaller package sizes and improved reliability, but challenges like delamination and outgassing limited their adoption. Advanced solutions like Die-Sized SAW Packaging (DSSP) and Thin-Film Acoustic Package (TFAP) further reduced package thickness and integrated copper pillar bumps (CPBs) for enhanced electrical performance. In contrast, BAW filters, particularly FBARs and SMRs, require hermetic sealing to protect the piezoelectric layer. Early solutions like Micro-Cap packaging faced issues with size and cost, while polymer-based non-hermetic packaging struggled with contamination. Advanced hermetic techniques, such as wafer-to-wafer bonding with silicon or glass caps and through-glass vias (TGVs), have improved performance but remain costly and complex. Overall, SAW WLP excels in cost and miniaturization, while BAW WLP offers superior performance for high-frequency applications, albeit at higher costs and complexity.

As shown in Table 1, WLP introduces distinct challenges and opportunities for SAW and BAW filters. SAW-based WLP achieves unparalleled miniaturization (<1 mm^2^) but faces parasitic losses at high frequencies, whereas BAW-WLP enables robust mmWave operation at the expense of process complexity and cost.

A key observation is the contradiction in the findings regarding the electrical performance of WLP solutions versus traditional packaging methods. While TGV-based packages show potential for superior performance, manufacturing defects, and variability remain concerns. While CPBs and TGVs offer significant advantages in terms of electrical performance and miniaturization, their scalability, cost implications, and limitations must be carefully considered. CPBs are highly scalable for high-volume manufacturing but require advanced lithography techniques for smaller bump pitches. TGVs, on the other hand, offer excellent scalability for high-frequency applications but are more expensive due to the cost of high-quality glass substrates and precision via formation. Both technologies face challenges in thermal management and mechanical stability, which need to be addressed for successful integration into real-world applications.

In conclusion, although significant strides have been made in advancing WLP technology for RF filters, further research is needed to bridge gaps in understanding the interplay between size reduction, performance, and reliability. Continued innovation in packaging techniques, scalable solutions, and material advancements are essential to meet the growing demand for miniaturized and high-performance components in next-generation wireless technologies. The ongoing evolution of WLP technologies is critical for the advancement in telecommunications, and this review serves as a foundation for future studies aimed at developing robust, efficient, and cost-effective RF filter solutions.

## Figures and Tables

**Table 1:** Comparison of SAW and BAW filters’ WLP solutions.

Metric	SAW WLP Solutions	BAW WLP Solutions
Size	Compact size (<1 mm^2^), direct wafer-level integration for ultra-miniaturized designs	A larger footprint (1–2 mm^2^) requires multilayer stacking or cavity structures.
Process Complexity	Low (CMOS-compatible SAW structures; no advanced micromachining required)	High (thin-film deposition, cavity etching, multilayer interconnects, and stress control)
WLP Reliability	Low	High
Temperature Stability	Moderate (TCF: −40 to −25 ppm/°C); WLP materials may exacerbate drift	Improved (TCF: −20 to −10 ppm/°C); drift mitigated via material matching
Reliability	High	Requires careful design

## Data Availability

Not applicable.
